# Polymorphism, Genetic Effect, and Association with Egg-Laying Performance of Chahua Chickens Matrix Metalloproteinases 13 Promoter

**DOI:** 10.3390/genes14071352

**Published:** 2023-06-27

**Authors:** Yanli Du, Changwei Cao, Yong Liu, Xiannian Zi, Yang He, Hongmei Shi, Jinbo Zhao, Changrong Ge, Kun Wang

**Affiliations:** 1 Yunnan Provincial Key Laboratory of Animal Nutrition and Feed, Yunnan Agricultural University, Kunming 650201, China; duyanligu@sina.com (Y.D.); lydzq05091025@163.com (Y.L.); 18826485012@163.com (Y.H.); 18388447069@163.com (H.S.); zhaojinbo312@163.com (J.Z.); gcrzal@126.com (C.G.); 2 College of Agronomy and Life Sciences, Kunming University, Kunming 650200, China; 3 Department of Food Science and Engineering, College of Biological Sciences, Southwest Forestry University, Kunming 650201, China; ccwylf1111@163.com

**Keywords:** Chahua chickens, age at first egg, ovary, MMP13, SNPs

## Abstract

Matrix metalloproteinases are a group of proteases involved in the regulation of ovarian follicular development and ovulation. Among the different MMPs, MMP13 is known to play an important role in reproduction. Therefore, this study aimed to screen the molecular genetic markers of the MMP13 gene that affect the egg-laying performance of Chahua chickens. Polymerase chain reaction (PCR) and sequencing were performed in the 5′ regulation region of the *MMP13* gene to detect loci significantly related to the egg-laying performance of Chahua chickens. A double fluorescence reporting system, quantitative reverse transcription PCR (RT-qPCR), and Western blotting were used to study whether gene expression was regulated by identified sites, providing a theoretical basis to improve egg production in Chahua chickens. The results revealed six single nucleotide polymorphisms (SNPs; A-1887T, T-1889C, A-1890T, T-2252C, T-2329C, and C-2360A) in the promoter region of the *MMP13* gene. Further analysis revealed that hens with T^-1890^-C^-1889^-T^-1887^/T^-1890^-C^-1889^-T^-1887^ (mutant type, MT) had an earlier age at first egg (AFE) than hens with A^-1890^-T^-1889^-A^-1887^/A^-1890^-T^-1889^-A^-1887^ (wild type, WT; *p* < 0.05). RT-qPCR showed that the relative expression level of the MMP13 gene in the ovarian tissues of individuals with the mutation was higher than that of individuals with the wild gene (*p* < 0.05). Western blot results confirmed higher levels of the MMP13 protein in MT ovaries compared to those in WT ovaries. Thus, this study suggests that mutation sites on the MMP13 promoter may affect gene expression. In conclusion, the *MMP13* gene in Chahua chickens may be significant for egg-laying performance, and the polymorphism in its promoter region could be used as a molecular marker to improve egg-laying performance.

## 1. Introduction

Chahua chicken is one of the native breeds of chickens grown in the unique tropical and subtropical parts of Yunnan, China. They are bred from Red Junglefowl (*Gallus gallus*). The main characteristics of this species are early sexual development and tender meat [1]. We analyzed the gene expression of high- and low-yielding Chinese Chahua laying chickens [2]; however, the exact mechanism remains elusive.

Poultry breeding is the most important economic aspect impacting poultry farming, as the selection of better breeds results in cheaper and faster meat production and maximum egg yield. The ovary represents a truly dynamic organ system characterized by structuring and restructuring events during the development of the ovarian follicle for ovulation [3,4]. Follicular development begins from the original follicle and grows about 400 times to form a mature follicle in domestic fowl. The development process includes recruitment, selection, dominance, growth, and maturation before ovulation [5]. Unlike mammals, the domestic fowl has only a single left ovary, containing follicles of various sizes and developmental stages [6]. Based on the follicles of various sizes and developmental stages, follicles are generally divided into small white follicles (SWFs), <2 mm in diameter, large white follicles (LWFs), 2~4 mm in diameter, small yellow follicles (SYFs), 4~8 mm in diameter, and hierarchical follicles (HFs) of F5 to F1, >8 mm in diameter [7]. To maintain continuous ovulation, an SYF is selected and entered into the HF layer each day, which then begins to grow rapidly and eventually differentiate [8,9]. It has been observed that follicular development and ovulation in the chicken’s ovary depend on the periodic degradation of the extracellular matrix and tissue reconstruction [10,11]. Studies have reported that the development of ovarian follicles is affected by FSH-mediated changes in the extracellular matrix, which can play an important role in the regulation of egg-laying performance [12,13]. Matrix metalloproteinases (MMPs) are known to regulate the extracellular matrix [14]. The elaborate control exerted by the MMP system can regulate a chicken’s follicle development and ovulation, which is essential to normal chicken’s ovarian follicle function [15].

*MMP13*, also known as collagenase-3, is a member of the MMP family and initiates the breakdown of the fibrillar collagens that form a key structural element of membranes. Recent studies have shown that MMP13 mRNA and/or protein expression increases with increased follicular maturation in the mature hen ovary follicles [16,17]. *MMP13* is also highly expressed in the ovaries of high-yielding Chinese Chahua-laying hens [2]. Studies have found that *MMP13* may be a genetic factor that affects chicken egg-laying performance [18]. From the studies mentioned above, it can be concluded that *MMP13* is significant for follicular development and reproduction. However, very little research has focused on the loci of the *MMP13* gene, especially noncoding loci, and their roles in regulating gene expression.

In this study, to verify whether *MMP13* could be a molecular genetic marker of egg-laying performance in Chahua chickens, we detected polymorphisms in the promoter of the *MMP13* gene that were associated with egg-laying performance and further analyzed transcriptional regulation. 

## 2. Materials and Methods

### 2.1. Animal Experimentation Ethical Statement

All animal experimental procedures were approved and guided by the Yunnan Agricultural University Animal Care and Use Committee (approval ID: YAUACUC01, publication date: 10 July 2013).

### 2.2. Experimental Animal, Sample Collection, and Preparation 

A total of 381 of 1848 Chahua chickens were randomly selected for polymorphism detection from Xishuangbanna Chahua Chicken Industrial Development Co., Ltd. They were given feed and water ad libitum under the same conditions of rearing and management. The egg-laying performance of each chicken was evaluated, including the age of the first egg (AFE), the body weight of the first egg (BWF), the total number of eggs at 30 weeks of age (EN30), the total number of eggs at 43 weeks of age (EN43), the egg weight at 30 weeks of age (EW30) and the egg weight at 43 weeks of age (EW43). Blood samples were collected from the subwing vein and stored at −20 °C. Genomic DNA was extracted from blood samples using the DNA Extraction Mini Kit (Tiangen, Beijing, China) and stored at −20 °C. The birds were treated according to the Animal Protection and Use Committee of Yunnan Agricultural University.

### 2.3. PCR Amplification and Sequencing

A pair of primers was designed to amplify a fragment of the promoter region of the *MMP13* gene (GenBank accession NC_006088.5). The specificity of the primers has been confirmed in the reference genome. The primer sequences are as follows: Forward–5′-AAGCTGAGTGCTGAAGAA-3′ and reverse–5′-TTCCACTCTGAATGTATC-3′. 2 × Taq PCR MasterMix (Biomed, Beijing, China) was used for PCR amplification; the total volume of the amplification system was 25 µL, including 2 × Taq PCR MasterMix (Biomed, Beijing, China)-12.5 µL, upstream and downstream primers-0.5 µL each, DNA template-1 µL (50 ng), and double-distilled H_2_O-11 µL. The following PCR conditions were followed: 95 °C for 5 min, 35 cycles of 94 °C for 30 s, 56 °C for 30 s, and 72 °C for 1 min and 30 s, and final extension at 72 °C for 10 min. Sequencing was performed with Applied Biosystem Inc. 3730XL automated DNA sequencer.

### 2.4. Construction of Fluorescent Dual Reporter Vector

Two genotypes (WT A-1890/T-1889/A-1887 and MT T-1890/C-1889/T-1887) were identified based on the sequencing results of the *MMP13* gene promoter, which were performed using a forward (5′-CCCGGGTGAATTTTCACTATTCAGCA-3′) and reverse primer (5′-AAGCTTTGTTGTTTCACTGTGGTCT-3′). The primers contained Hind III and Sma Ι restriction sites (underlined nucleotides). The 5′-regulatory region from −2301 to +1 bp of the chicken *MMP13* gene, where +1 is the transcription initiation site, was cloned. The PCR fragments containing each allele were cloned into the pGL3 Luciferase Reporter Vector (pGL3-Basic vector; Promega, Madison, WI, USA).

### 2.5. Double Fluorescence Report Detection

The experiments were carried out on 293T human kidney epithelial (HEK) cells, which were provided by the Kunming Institute of Zoology. Chinese Academy of Sciences. These cells were derived from the insertion of T antigen in 293HEK cells. Subsequently, they were cultured in Dulbecco’s modified eagle medium (DMEM; 10% fetal bovine serum and 1% double-antibody) at 37 °C and 5% CO_2_. When cells were in good condition, they were transferred to 48-well plates, and 60,000–70,000 cells were cultured in each well for 24 h. For cotransfection of cells, lipofectamine-2000 (Invitrogen, Waltham, MA, USA) was used as the transfection reagent. The constructed reporter vector and empty vector were added to the wells at a concentration of 0.05 µg/well, and transfection was carried out in strict accordance with the instructions. The experiments were carried out in triplicate. After 48 h, the protein was extracted from the lysed cells, and luciferase activity was detected on the GloMax Discover Microplate Reader (96-well) using a fluorescence double reporter assay kit. 

### 2.6. RT-qPCR

The Takara total RNA extraction kit (Takara, Beijing, China) was used to extract RNA from the 8 WT and 8 MT Chahua chickens’ ovarian tissue randomly selected, and the purity of the RNA was determined using a NanoDrop 2000 spectrophotometer. Agilent 2100 bioanalyzer was used to detect RNA integrity. The degradation of RNA was detected by 1% agarose gel electrophoresis. The PrimeScript RT Reagent Kit with gDNA Eraser (Takara, Beijing, China) was used for cDNA synthesis. First, any DNA contamination in the RNA was removed, and the volume of this reaction system was 10 µL, including total RNA-1 µg, 5 × gDNA eraser buffer-2 µL, gDNA eraser-1 µL, and RNase-free dH_2_O-make upto10 µL. Then reverse transcription to cDNA required 20 µL of reaction liquid, including 5 × PrimeScript buffer-4 µL, PrimeScript RT enzyme mix-1 µL, RT primer mix-1 µL, above reaction liquid (from the DNA removal step)-10 µL, and RNase-free dH_2_O-4 µL. The reverse transcription reaction was carried out at 37 °C for 15 min and 85 °C for 5 s. The PCR reaction system was 20 µL, including SYBR Premix Ex TapTM Green II-10 µL, forward primer-2 µL, reverse primer-2 µL, cDNA template-1 µL, and RNase-free dH_2_O-5 µL. The PCR reaction conditions were 94 °C for 30 s to activate the reaction and 94 °C for 15 s, 56 °C for 30 s and 72 °C for 30 s for 40 cycles. The β-actin gene was used as an internal reference, and the relative expression of RNA was calculated as follows. Relative expression = 2^−ΔΔ*C*T^.

### 2.7. Western Blot

The total protein was extracted from 4 WT and 4 MT Chahua chickens’ ovarian tissue randomly selected using a rapid cell lysate kit (Solarbio, Beijing, China). Protein concentration was quantified using the BCA protein quantification kit (Tiangen, Beijing, China). The proteins were separated by SDS-PAGE gel electrophoresis under denaturing and non-reducing conditions and transferred to a polyvinylidene fluoride membrane (PVDF). After the addition of a 5% bovine serum albumin/TBST solution, the membrane was agitated for 1 h. Mouse monoclonal antibody anti-MMP-13 (Thermo, New York, NY, USA, 1:1000) was added, and the membrane was agitated for 30 min, followed by overnight incubation at 4 °C. The membrane was washed 3 times with TBST for 10 min each, followed by the addition of goat anti-mouse immunoglobulin G antibody (Thermo, New York, NY, USA, 1:1000) and incubation in a shaker for 1 h. The 3 TBST washes were performed again for 10 min each. The PVDF membrane was exposed to chemiluminescence to obtain the experimental results. The intensities of the blots were quantified using ImageJ software [19].

### 2.8. Data Statistics and Analysis

Pearson correlation analysis was conducted for egg-laying traits using SPSS 25.0 software. The frequency of alleles and genotypes, genetic homozygosity (Ho), genetic heterozygosity (He), effective allele (Ne), polymorphism information content (PIC), and genetic balance of the Hardy-Weinberg population were detected using the Pobgene software. SHEsis software was used to perform linkage disequilibrium analysis and haplotype analysis. The linkage disequilibrium coefficient D and the correlation coefficient R^2^ were used to determine the linkage disequilibrium at the mutation sites. The linkage imbalance occurs when D > 0.75 and R^2^ > 0.33. The general linear model using SPSS 25.0 software was employed to analyze the association between gene mutation sites and egg-laying performance, as follows:

Y*_ij_* = u + G*_i_* + e*_ij_*

where Y*_ij_* represents the phenotypic value of the trait or the reproductive level, u represents the overall mean, G*_i_* represents the genotype fixed effect, and e*_ij_* represents the random error. The *p*-values were corrected using multiple testing, and the corrected *p*-values < 0.05 were used as the threshold for detecting the significant differential expression.

For qRT-PCR analysis, Western blotting analysis, and luciferase assay, differences between groups were evaluated by *t*-test (*p* < 0.05). All expressions were repeated at least 3 times, and all data were presented as mean ± SEM. 

## 3. Results

### 3.1. Correlation Analysis of Egg-Laying Performance in Chahua Chickens

There are 12 pairs of traits that show significant correlations (*p* < 0.05; Table 1). There was a significant negative correlation between BWF and AFE. BWF was positively correlated with EW30, EN30, EN30-EN43, EW43, and EN43, and AFE was significantly negatively correlated with EN30 and EN43. A significant positive correlation was observed between EW30 and EW43 and between EN30, EN30-EN43, and EN43 (*p* < 0.05). 

### 3.2. Polymorphisms of the 5′ Regulatory Region in Chahua Chickens’ MMP13 Gene

**Figure 1:**
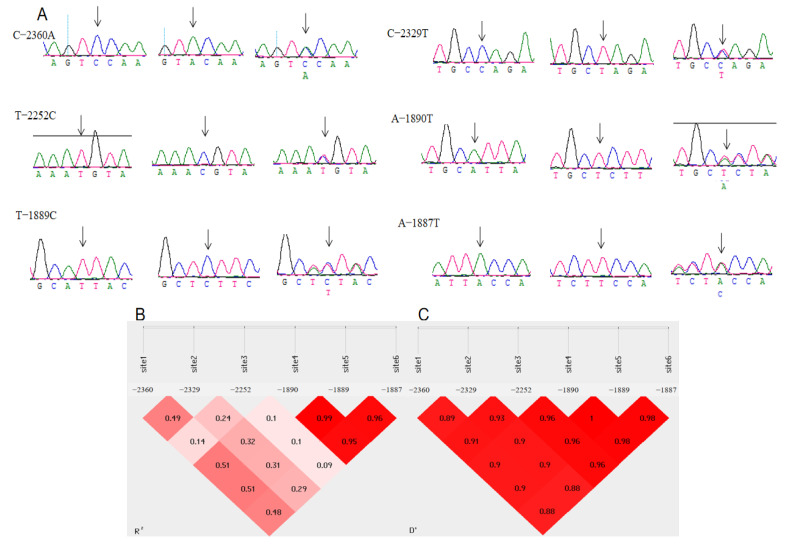
Six single nucleotide polymorphisms identified in the promoter region of the *MMP13* gene (**A**), The R^2^ map (**B**), and the D map (**C**) of linkage disequilibrium in chickens. With D > 0.75 and R^2^ > 0.33, there is a linkage disequilibrium, the arrow points to the mutation site.

The 5′ regulatory region of the *MMP13* gene of Chahua chickens was sequenced to detect the mutation sites. The base A of the start codon (ATG) of the gene was set as +1, and the first base upstream of the start codon was set as-1 and counted successively. According to the sequencing results (Figure 1), a total of six SNPs were found in the 5′ regulatory region of the *MMP13* gene in Chahua chickens, which were located upstream at 1887 and 1889, 1890, 2252, 2329, and 2360 bases. They are named g.-2360C > A, g.-2329T > C, g.-2252T > C, g.-1890A > T, g.-1889T > C, and g.-1887A > T. Since there were six loci in the *MMP13* gene, we performed a linkage disequilibrium analysis, and according to the results, the A-1890T, T-1889C, and A-1887T loci exhibited complete linkage. The gene and genotypes frequencies of the six SNPs of the *MMP13* gene (g.-2360C > A, g.-2329T > C, g.-2252T > C, g.-1890A > T, g.-1889T > C, and g.-1887A > T) are shown in Table S1. The dominant alleles observed at the six loci were A, T, C, A, C, and A, respectively, which were detected by the Hardy-Weinberg test and were observed to be in equilibrium (*p* > 0.05). The PIC of the six SNPs of the *MMP13* gene (g.-2360C > A, g.-2329T > C, g.-2252T > C, g.-1890A > T, g.-1889T > C, and g.-1887A > T) are shown in Table S2. Except for g.-2252T > C, which exhibited low polymorphism, the rest demonstrated moderate polymorphism. The Ho, He and Ne values are also shown in Table S2. (The sequence has been submitted to the NCBI, the GeneBank accession number: OK356868).

### 3.3. Analysis of the Association of Different Genotypes of the MMP13 Gene with the Egg-Laying Performance of Chahua Chickens

Association analysis was performed to evaluate the association between phenotypic data of the laying traits of Chahua chickens and different genotypes of *MMP13* (Table 2). The results indicate that site g.-2360C > A was significantly associated with the BWF (*p* < 0.0008) and EN30 (*p* < 0.0074). In addition, the EN30 of the AA genotype was higher than that of the CC genotype (*p* < 0.0074); the BWF of the AA genotype was heavier than that of the CC genotype (*p* < 0.0008). Analysis of site g.-1890A > T revealed that it was significantly associated with AFE (*p* < 0.0061) and EW43 (*p* < 0.0076). The AFE of the TT genotype was earlier than that of the AA genotype (*p* < 0.0061); EW43 of the TT genotype was heavier than that of the AA genotype (*p* < 0.0076). The site g.-1889T > C was significantly associated with AFE (*p* < 0.0031) and EW43 (*p* < 0.0064). The AFE of the CC genotype was earlier than the TT genotype (*p* < 0.0031); the EW43 of the CC genotype was heavier than that of the TT genotype (*p* < 0.0064). Analysis of the site g.-1887A > T was significantly associated with AFE (*p* < 0.0078) and EW43 (*p* < 0.0069). The AFE of the TT genotype was earlier than that of the AA genotype (*p* < 0.0078), and the EW43 of the TT genotype was heavier than that of the AA genotype (*p* < 0.0029). 

### 3.4. Analysis of the Association of Different Haplotypes of the MMP13 Gene with Egg-Laying Performance of Chahua Chicken 

To analyze the associations of SNPs in the chicken *MMP13* promoter region with egg-laying performance, haplotypes were constructed using the three SNPs A-1887T, T-1889C, and A-1890T. Two haplotypes of A (T-1890/C-1889/T-1887) and B (A-1890/T-1889/A-1887) were detected in the Chahua chicken population. The association of haplotypes A and B with the egg-laying performance of Chahua chickens should be analyzed. The results indicated that the hens with haplotype A had an earlier AFE than those with haplotype B (*p* < 0.0053). Furthermore, hens with the A haplotype exhibited heavier BWF than those with haplotype B (*p* < 0.00063; Table 3). 

### 3.5. The Effect of SNPs on the Promoter Activity of the MMP13 Gene

**Figure 2:**
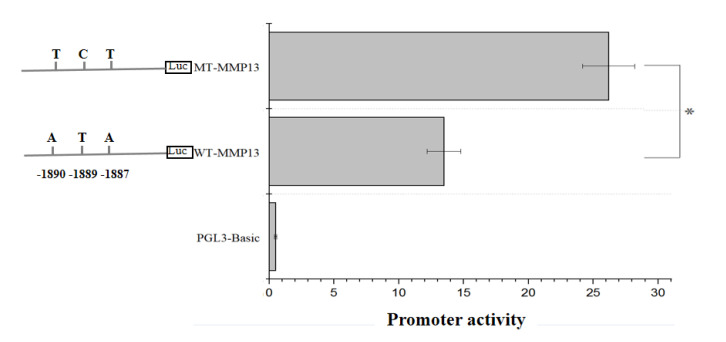
Results of dual fluorescence reporter detection and expression vector analysis In the figure, pGL3-Basic represents the empty vector, WT-MMP13 represents the reporter vector containing A-1890/T-1889/A-1887 polymorphisms, and MT-MMP13 represents the reporter vector containing T-1890/C-1889/T-1887 polymorphisms, and the X-axis represents the relative promoter activity of the vector, * *p* < 0.05.

We analyzed the possible functions of four SNPs (g.-2360C > A, g.-1890A > T, g.-1889T > C, and g.-1887A > T) that were significantly associated with an egg-laying performance by association analysis. Since these four SNPs are located in the promoter region of the gene, we used the online tool JASPAR (http://jaspar.genereg.net/; accessed on 16 April 2022) to search for transcription factors that may bind to this region. It was found that the EHF specifically binds to regions that exhibit complete linkage (g.-1890A > T, g.-1889T > C, and g.-1887A > T). We tested whether the A-1890/T-1889/A-1887 and T-1890/C-1889/T-1887 polymorphisms (designated as WT-MMP13 and MT-MMP13) regulate gene promoter activity by a dual fluorescence reporter system. The results indicated that compared to the empty vector, the two reporter vectors, pGL3-WT and pGL3-MT, showed significant promoter activity. The promoter activity of pGL3-MT with the T-1890/C-1889/T-1887 polymorphisms was significantly higher than that of pGL3-WT with the A-1890/T-1889/A-1887 polymorphisms (Figure 2; *p* < 0.05). These results suggest that site mutations in this region may affect promoter activity.

### 3.6. Analysis of the MMP13 Gene Expression Level 

**Figure 3:**
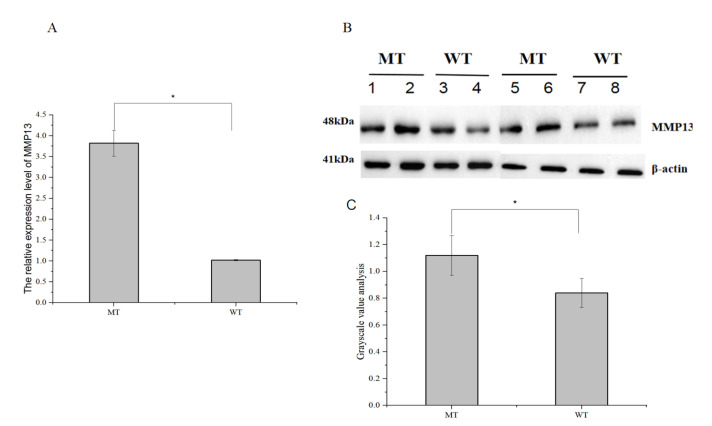
Analysis of mRNA (**A**), protein expression levels (**B**) and Gray value analysis (**C**) of the *MMP13* gene promoter haplotype A-1890/T-1889/A-1887 (WT) and T-1890/C-1889/T-1887 (MT) in Chahua chickens, * *p* < 0.05.

To study the effect of mutations occurring at different sites of the *MMP13* promoter on gene expression, quantitative detection of the expression level of the *MMP13* gene was carried out. The results showed that the relative expression level of the *MMP13* gene in the ovarian tissues of the MT individuals (T-1890/C-1889/T-1887) was higher than that of the WT individuals (A-1890/T-1889/A-1887). The results of Western blotting were consistent with the above results, i.e., the protein expression level of the *MMP13* gene in MT was higher than in WT (Figure 3). Together, all the results further verified our speculation that the promoter mutation site might affect the expression of the *MMP13* gene.

## 4. Discussion

We conducted a correlation analysis on the laying traits of 1848 Chahua chickens. There were twelve pairs of traits with significant correlation (*p* < 0.05; Table 1). The BWF of the Chahua chicken was negatively correlated with its AFE. A study conducted on Jiuyuan black chickens showed that BWF was significantly negatively correlated with AFE, which was consistent with the results of this study [20]. The AFE of Chahua chickens was negatively correlated with EN30 and EN43. However, the AFE of Chahua chickens was no significant correlation with EN30–43. Since the rate of ovarian maturation determines the AFE of the chicken, the faster the ovary matures, the earlier the AFE of the chicken, and the higher the early egg production [21]. Based on this, it can be concluded that AFE may be an indicator of early egg production. Furthermore, EW30 was significantly negatively correlated with EN30 and EN43, while EN30 was significantly negatively correlated with EW43. Thus, egg weight and number are important factors to consider in breeding.

During the follicular growth of the chicken ovary, the extracellular matrix continuously reconstructs the interactions between cells and cell-matrix interactions; this process depends on the regulation of MMP activity by endogenous inhibitors called tissue inhibitors of metalloproteinases (TIMPs) [22]. Fine regulation of the MMP system is known to regulate follicular development and ovulation [17]. Previous studies have characterized the expression and molecular mechanism of chicken *MMP2* and *MMP9* in ovarian follicles [10,15]. A study reported an increase in the expression of MMP13 mRNA in rat ovaries after 36–48 h of gonadotropin treatment [23]. Therefore, gonadotropin may also be a regulator of MMP expression in chicken ovarian follicles [24]. In chickens, *MMP13* has effects on ovarian follicle development and ovulation [11]. Therefore, in this study, we identified SNPs associated with egg-laying performance and analyzed the regulation of chicken *MMP13*. In our study, four SNPs (g.-2360C > A, g.-1890A > T, g.-1889T > C, and g.-1887A > T) were identified in the upstream region of the *MMP13* promoter that were significantly associated with egg-laying performance. A previous study has reported that *MMP13* is highly expressed in the ovaries of sexually mature chickens, and two SNPs (g.-1356A > G and g-1079C > T) are significantly associated with egg-laying performance [25]. Therefore, the *MMP13* gene may be closely related to egg-laying performance in chickens. We analyzed the possible functions of the significantly associated SNPs (g.-2360C > A, g.-1890A > T, g.-1889T > C, and g.-1887A > T) and evaluated the binding of transcription factor EHF to the complete linkage region (g.-1890A > T, g.-1889T > C, and g.-1887A > T) through JASPAR. EHF, also known as epithelial specificity conversion factor 3 (epithelium-specific ETS factor family member 3 [ESE3]), widely exists in the nucleus and belongs to the transcriptional regulatory factor family. It can form a transcription complex, alone or with other molecules, thus enhancing or inhibiting downstream gene transcription; also, it is known to affect cell proliferation, development, differentiation, apoptosis, and aging [26]. The functions of EHF depend mainly on its ETS and PNT domains. The ETS domain contains 85 amino acids and forms a winged-helix-turn-helix DNA binding motif, which regulates the transcription of downstream genes by specifically binding to the promoter region of a target gene, leading to changes in cell biological functions [27]. EHF generally binds to the promoter region 5′-GGAA/T-3′ to bring about the transcriptional regulation of downstream genes, but it can also bind to non-classical regions when interacting with other transcription factors, such as 5′-GGAG-3′ [27]. The PNT domain contains 80 amino acids and is located at the N-terminal. Its main functions are to mediate protein-protein interactions, kinase docking, RNA binding, lipid molecular interactions, and transcriptional activation [28,29]. Two haplotypes of A (T-1890/C-1889/T-1887) and B (A-1890/T-1889/A-1887) were detected in the Chahua chicken population. Haplotypes of A (T-1890/C-1889/T-1887) have earlier AFEs in the Chahua chicken population. Furthermore, the results of the luciferase assay indicated that the *MMP13* gene promoter harboring T-1890/C-1889/T-1887 has higher transcriptional activity than that of the promoter of A-1890/T-1889/A-1887 in Chahua chickens. This confirms that the mutation site affected the promoter activity of the gene. 

Next, to study whether the SNPs (g.-1890A > T, g.-1889T > C, and g.-1887A > T) of the *MMP13* gene promoter affect gene expression, individuals with A-1890/T-1889/A-1887 and T-1890/C-1889/T-1887 were selected for quantitative detection of *MMP13* relative expression level. The results indicated that the T-1890/C-1889/T-1887 ovaries had higher expression levels of the *MMP13* gene compared to the A-1890/T-1889/A-1887 ovaries. The results of Western blot were consistent with the above results, i.e., MMP13 protein expression levels were higher in T-1890/C-1889/T-1887 tissues than in A-1890/T-1889/A-1887 tissues. Therefore, it is suggested that the mutation site in the promoter may affect *MMP13* gene expression. Based on this, we speculated that these SNPs affected the expression of the *MMP13* gene in the ovarian tissue of Chahua chickens by changing the affinity between the promoter region and different transcriptional activators or suppressors, revealing the regulatory mechanism of egg-laying in Chahua chickens. Additionally, functional verification of the SNPs of the *MMP13* gene (A-1890T, T-1889C, A-1887T) would lead to their use as molecular genetic markers for laying traits in Chahua chickens.

## 5. Conclusions

In conclusion, six SNPs of the *MMP13* gene were identified by sequencing and genotyping in Chahua chicken populations. Hens with the T-1890/C-1889/T-1887 haplotype had an earlier AFE than those with the A-1890/T-1889/A-1887 haplotype and exhibited higher transcriptional activity, which was confirmed by luciferase assay. The expression levels of the *MMP13* gene mRNA and protein in the ovary tissues of individuals with T-1890/C-1889/T-1887 were higher than those of individuals with A-1890/T-1889/A-1887. These results collectively suggest that *MMP13* may play an important role in egg-laying and that polymorphisms in its promoter region could be used as molecular markers to improve egg-laying performance in chicken breeding.

## Figures and Tables

**Table 1:** Correlation analysis of the egg-laying performance of Chahua chickens.

	BWF	AFE	EW 30	EN 30	EN30–EN43	EW 43	EN 43
BWF	1						
AFE	−0.324 **	1					
EW 30	0.302 **	0.082	1				
EN 30	0.194 **	−0.577 **	−0.09	1			
EN30–EN43	0.313 **	−0.092	−0.025	0.742 **	1		
EW 43	0.333 **	−0.043	0.598 **	−0.106	−0.021	1	
EN 43	0.119 *	−0.399 **	−0.019	0.902 **	0.845 **	−0.081	1

Note: BWF: the body weight at the first egg, AFE: the age at the first egg, EN30: the total number of eggs at 30 weeks of age, EW30: the egg weight at 30 weeks of age, EN43:the total number of eggs at 43 weeks of age, EN30-EN43: the total number of eggs at 30–43 weeks of age, EW43: the egg weight at 43 weeks of age, * *p* < 0.05, ** *p* < 0.01.

**Table 2:** Analysis of the association between different genotypes of the *MMP13* gene and egg-laying performance of Chahua chickens.

SNPs	Quantity	Genotype	BWF (g)	AFE (Days)	EW 30 (g)	EN 30 (Count)	EW 43 (g)	EN 43 (Count)
2360C > A	381	AA	1057.56 ± 12.69 ^a^	146.45 ± 1.37	39.74 ± 0.42	56.91 ± 1.54 ^a^	42.93 ± 0.42	84.43 ± 3.64
		AC	1027.75 ± 7.91 ^ab^	148.95 ± 0.82	37.12 ± 0.41	52.88 ± 1.07 ^ab^	41.48 ± 0.37	89.13 ± 2.38
		CC	1024.51 ± 10.82 ^b^	150.29 ± 1.45	37.47 ± 0.47	52.08 ± 1.83 ^b^	42.31 ± 0.71	87.2 ± 3.76
2329T > C	381	CC	1062.45 ± 10.32	146.11 ± 1.05	38.94 ± 0.33	55.49 ± 1.26	42.4 ± 0.356	88.32 ± 2.804
		CT	1020.86 ± 7.74	150.06 ± 0.88	37.19 ± 0.46	52.17 ± 1.27	41.71 ± 0.421	87.21 ± 2.607
		TT	996.46 ± 12.24	150.55 ± 1.85	36.93 ± 0.61	52.89 ± 2.48	42.01 ± 0.975	85.75 ± 4.628
2252T > C	381	CC	1040.23 ± 6.91	147.41 ± 0.72	38.14 ± 0.27	54.95 ± 0.88	41.99 ± 0.29	89.02 ± 1.952
		CT	1018.47 ± 10.64	151.44 ± 1.38	36.91 ± 0.82	50.48 ± 1.85	42.31 ± 0.65	83.98 ± 4.10
		TT	1004.78 ± 28.98	156.64 ± 3.77	37.22 ± 0.53	45.17 ± 5.61	41.56 ± 1.79	78.27 ± 10.50
1890A > T	381	AA	1015.80 ± 9.55	151.02 ± 1.09 ^a^	36.60 ± 0.59	52.18 ± 1.35	41.64 ± 0.46 ^b^	83.99 ± 3.09
		AT	1037.91 ± 8.53	147.77 ± 0.91 ^ab^	37.96 ± 0.27	54.28 ± 1.12	41.91 ± 0.39 ^ab^	89.57 ± 2.48
		TT	1065.36 ± 13.09	146.31 ± 1.52 ^b^	40.09 ± 0.46	54.82 ± 2.1	43.24 ± 0.52 ^a^	89.03 ± 4.07
1889T > C	381	TT	1017.48 ± 9.47	151.03 ± 1.08 ^a^	36.57 ± 0.58	51.92 ± 1.34	41.6 ± 0.45 ^b^	84.05 ± 3.05
		TC	1036.95 ± 8.61	147.74 ± 0.92 ^ab^	38.02 ± 0.27	54.49 ± 1.13	41.95 ± 0.40 ^ab^	89.58 ± 2.50
		CC	1065.36 ± 13.09	146.21 ± 1.52 ^b^	40.09 ± 0.46	54.82 ± 2.11	43.24 ± 0.52 ^a^	89.03 ± 4.01
1887A > T	381	AA	1016.26 ± 9.37	151.07 ± 1.06 ^a^	36.61 ± 0.57	52.13 ± 1.33	41.6 ± 0.45 ^b^	83.73 ± 3.03
		AT	1037.69 ± 8.76	147.78 ± 0.94 ^ab^	38.01 ± 0.27	54.49 ± 1.14	41.95 ± 0.40 ^ab^	90.09 ± 2.53
		TT	1065.52941 ± 12.70	145.74 ± 1.48 ^b^	40.09 ± 0.46	54.49 ± 2.05	43.24 ± 0.52 ^a^	88.34 ± 4.032

Note: BWF: body weight at first egg, AFE: age at first egg, EN30: total number of eggs at 30 weeks of age, EW30: egg weight at 30 weeks of age, EN43: total number of eggs at 43 weeks of age and EW43: egg weight at 43 weeks of age; Means with different lower case letters within the same column are significantly different (*p* < 0.05).

**Table 3:** Analysis of the association of the haplotype of *MMP13* from three sites (A-1890T, T-1889C, and A-1887T) haplotype of *MMP13* with egg-laying performance.

Diplotype	BWF	AFE	EW 30	EN 30	EW 43	EN 43
A	1062.46 ± 12.96 ^a^	145.62 ± 1.49 b	40.11 ± 0.46	55.17 ± 2.11	43.24 ± 0.44	90.67 ± 3.02
B	1015.30 ± 9.67 ^b^	150.94 ± 0.89 a	38.19 ± 0.47	52.41 ± 1.36	41.11 ± 0.32	87.59 ± 3.01

Note: BWF: body weight at first egg, AFE: age at first egg, EN30: total number of eggs at 30 weeks of age, EW30: egg weight at 30 weeks of age, EN43: total number of eggs at 43 weeks of age and EW43: egg weight at 43 weeks of age; Means with different lower case letters within the same column are significantly different (*p* < 0.05).

## Data Availability

The data presented in this study are available on request from the corresponding author. The data are not publicly available due to privacy.

## Supplementary Materials

The following supporting information can be downloaded at: https://www.mdpi.com/article/10.3390/genes14071352/s1, Table S1: Allele and genotype frequency of *MMP13*, Table S2: SNPs parameters of MMP13 in Chahua chickens.

## References

[1] Li J., Zhao Z., Xiang D., Zhang B., Ning T., Duan T., Rao J., Yang L., Zhang X., Xiong F. (2018). Expression of APOB, ADFP and FATP1 and their correlation with fat deposition in Yunnan’s top six famous chicken breeds. Br. Poult. Sci..

[2] Du Y., Liu L., Liu Y., Wang K., Shi H., He Y., Long Y., Sun D., Wu H., Zi X. (2022). Ovary transcriptome profiling in high- and low-yielding Chinese Chahua laying chickens. Czech J. Anim. Sci..

[3] Kim M.H., Seo D.S., Ko Y. (2004). Relationship Between Egg Productivity and Insulin-Like Growth Factor-I Genotypes in Korean Native Ogol Chickens. Poult. Sci..

[4] Evans A.C. (2003). Characteristics of Ovarian Follicle Development in Domestic Animals. Reprod. Domest. Anim..

[5] Regan S.L., Knight P.G., Yovich J.L., Leung Y., Arfuso F., Dharmarajan A. (2018). Involvement of Bone Morphogenetic Proteins (BMP) in the Regulation of Ovarian Function. Vitam. Horm..

[6] Gilbert A.B., Perry M.M., Waddington D., Hardie M.A. (1983). Role of atresia in establishing the follicular hierarchy in the ovary of the domestic hen (*Gallus domesticus*). Reproduction.

[7] Hocking P.M. (2009). Biology of breeding poultry. Poultry Science Symposium Series Bodmin.

[8] Onagbesan O., Bruggeman V., Decuypere E. (2009). Intra-ovarian growth factors regulating ovarian function in avian species: A review. Anim. Reprod. Sci..

[9] Ghanem K., Johnson A. (2018). Follicle dynamics and granulosa cell differentiation in the turkey hen ovary. Poult. Sci..

[10] Hrabia A., Wolak D., Kwaśniewska M., Kieronska A., Socha J.K., Sechman A. (2018). Expression of gelatinases (MMP-2 and MMP-9) and tissue inhibitors of metalloproteinases (TIMP-2 and TIMP-3) in the chicken ovary in relation to follicle development and atresia. Theriogenology.

[11] Wolak D., Sechman A., Hrabia A. (2021). Effect of eCG treatment on gene expression of selected matrix metalloproteinases (MMP-2, MMP-7, MMP-9, MMP-10, and MMP-13) and the tissue inhibitors of metalloproteinases (TIMP-2 and TIMP-3) in the chicken ovary. Anim. Reprod. Sci..

[12] Ingman W., Owens P., Armstrong D. (2000). Differential regulation by FSH and IGF-I of extracellular matrix IGFBP-5 in bovine granulosa cells: Effect of association with the oocyte. Mol. Cell. Endocrinol..

[13] Huet C., Pisselet C., Mandon-Pépin B., Monget P., Monniaux D. (2001). Extracellular matrix regulates ovine granulosa cell survival, proliferation and steroidogenesis: Relationships between cell shape and function. J. Endocrinol..

[14] Curry T.E., Osteen K.G. (2003). The matrix metalloproteinase system: Changes, regulation, and impact throughout the ovarian and uterine reproductive cycle. Endocr. Rev..

[15] Leśniak-Walentyn A., Hrabia A. (2017). Expression and localization of matrix metalloproteinases (MMP-2, -7, -9) and their tissue inhibitors (TIMP-2, -3) in the chicken oviduct during pause in laying induced by tamoxifen. Theriogenology.

[16] Hrabia A. (2021). Matrix Metalloproteinases (MMPs) and Inhibitors of MMPs in the Avian Reproductive System: An Overview. Int. J. Mol. Sci..

[17] Wolak D., Hrabia A. (2020). Alternations in the expression of selected matrix metalloproteinases (MMP-2, -9, -10, and −13) and their tissue inhibitors (TIMP-2 and -3) and MMP-2 and -9 activity in the chicken ovary during pause in laying induced by fasting. Theriogenology.

[18] Du Y., Liu L., He Y., Dou T., Jia J., Ge C. (2020). Endocrine and genetic factors affecting egg laying performance in chickens: A review. Br. Poult. Sci..

[19] Taylor S.C., Posch A. (2014). The Design of a Quantitative Western Blot Experiment. BioMed Res. Int..

[20] Liu J., Miao X.M., Li F.G., Zhu Q., Wang Y.Y., Yin H.D., Shu G., Ye L., Zhao X.L. (2020). The curves fitting for laying rate and cumulative egg production, and the correlation analysis for laying performance of Jiuyuan Black Chickens. J. Yunnan Agric. Univ..

[21] Zou K., Asiamah C.A., Lu L.-L., Liu Y., Pan Y., Chen T., Zhao Z., Su Y. (2020). Ovarian transcriptomic analysis and follicular development of Leizhou black duck. Poult. Sci..

[22] Zhou F., Shi L.-B., Zhang S.-Y. (2017). Ovarian Fibrosis: A Phenomenon of Concern. Chin. Med. J..

[23] Cooke R.G.I., Nothnick W.B., Komar C., Burns P., Curry T.E. (1999). Collagenase and Gelatinase Messenger Ribonucleic Acid Expression and Activity During Follicular Development in the Rat Ovary1. Biol. Reprod..

[24] Zhu G., Kang L., Wei Q., Cui X., Wang S., Chen Y., Jiang Y. (2014). Expression and Regulation of MMP1, MMP3, and MMP9 in the Chicken Ovary in Response to Gonadotropins, Sex Hormones, and TGFB11. Biol. Reprod..

[25] Yuan Z., Chen Y., Chen Q., Guo M., Kang L., Zhu G., Jiang Y. (2016). Characterization of Chicken *MMP13* Expression and Genetic Effect on Egg Production Traits of Its Promoter Polymorphisms. G3 Genes|Genomes|Genet..

[26] Luk I.Y., Reehorst C.M., Mariadason J.M. (2018). ELF3, ELF5, EHF and SPDEF Transcription Factors in Tissue Homeostasis and Cancer. Molecules.

[27] Kameyama N., Kobayashi K., Shimizu S., Yamasaki Y., Endo M., Hashimoto M., Furihata T., Chiba K. (2016). Involvement of ESE-3, epithelial-specific ETS factor family member 3, in transactivation of the ABCB1 gene via pregnane X receptor in intestine-derived LS180 cells but not in liver-derived HepG2 cells. Drug Metab. Pharmacokinet..

[28] Hollenhorst P.C., Ferris M.W., Hull M.A., Chae H., Kim S., Graves B.J. (2011). Oncogenic ETS proteins mimic activated RAS/MAPK signaling in prostate cells. Genes Dev..

[29] Madison B.J., Clark K.A., Bhachech N., Hollenhorst P.C., Graves B.J., Currie S.L. (2018). Electrostatic repulsion causes anticooperative DNA binding between tumor suppressor ETS transcription factors and JUN–FOS at composite DNA sites. J. Biol. Chem..

