# Supplementary material for: Polymorphism, Genetic Effect, and Association with Egg-Laying Performance of Chahua Chickens Matrix Metalloproteinases 13 Promoter
Source: Genes (Basel). 2023 Jun 27;14(7):1352. doi: 10.3390/genes14071352 (PMC10379211; doi:10.3390/genes14071352)
Supplement: Supplementary file 1 [file genes-14-01352-s001.zip › Table S1.pdf]

genes

MDPI

Table S1. Allele and genotype frequency of MMP13.

Site	Sample	Genotype Frequency			Allele Frequency		χ2	*p* Value
2360C>A	381	CC	AC	AA	A	C		
		0.202 (77)	0.538 (205)	0.276 (105)	0.524	0.476	2.95	0.09
2329T>C	381	TT	TC	CC	T	C		
		0.147 (56)	0.451 (172)	0.402 (153)	0.543	0.457	0.045	0.83
2252T>C	381	CC	TC	TT	C	T		
		0.756 (289)	0.197 (75)	0.044 (17)	0.864	0.136	2.91	0.087
1890A>T	381	AA	AT	TT	A	T		
		0.336 (128)	0.491 (187)	0.073 (66)	0.581	0.419	0.018	0.89
1889T>C	381	TT	TC	CC	T	C		
		0.341 (130)	0.486 (185)	0.173 (66)	0.416	0.584	0.0014	0.97
1887A>T	381	AA	AT	TT	A	T		
		0.346 (132)	0.475 (181)	1.789 (68)	0.585	0.415	0.28	0.59

The numbers in parentheses represent the number of samples.
