# Supplementary material for: Polymorphism, Genetic Effect, and Association with Egg-Laying Performance of Chahua Chickens Matrix Metalloproteinases 13 Promoter
Source: Genes (Basel). 2023 Jun 27;14(7):1352. doi: 10.3390/genes14071352 (PMC10379211; doi:10.3390/genes14071352)
Supplement: Supplementary file 1 [file genes-14-01352-s001.zip › Table S2.pdf]

genes

Table S2. SNPs parameters of MMP13 in Chahua chickens.

Site	Sample	Ho	He	Ne	PIC
-2360 C/A	381	0.456	0.544	2.19	0.37
-2329 T/C	381	0.543	0.457	1.84	0.36
-2252 T/C	381	0.802	0.198	1.25	0.21
-1890 A/T	381	0.509	0.491	1.96	0.37
-1889 T/C	381	0.653	0.486	1.53	0.37
-1887 A/T	381	0.496	0.472	2.02	0.37

Abbreviations: Homozygosity-Ho, Heterozygosity-He, Effective Allele Number-Ne, Polymorphism Information Content-PIC.
